# Back to the Future—A 50-Year Dive into Embryo Implantation Research: Cell Biological Paradox, Epithelial Cell Polarity, and EMT

**DOI:** 10.3390/biom16020293

**Published:** 2026-02-13

**Authors:** Hans-Werner Denker

**Affiliations:** Institut für Anatomie, Universität Duisburg-Essen, 45147 Essen, Germany; hans-werner.denker@uni-due.de

**Keywords:** embryo implantation, epithelial cell polarity, EMT, signalling, in vitro models

## Abstract

Embryo implantation presents a cell biological paradox: contact formation between the trophoblast of the blastocyst and the epithelial lining of the endometrium contradicts typical epithelial cell behaviour, as does the subsequent invasion needed for placenta formation in most species (including the human). Explaining this conundrum became a challenge for investigation since its recognition about 40 years ago and it receives increasing interest because implantation failure appears to be a major cause for low success in assisted reproduction. The present article reviews the main findings that have directed attention of researchers on epithelial cell polarity and on the theoretical concept of epithelial–mesenchymal transition (EMT). Apart from trophoblast attachment competence, a special focus is on endometrial receptivity. Comparison with epithelial fusion processes (EFPs) in development and with tumour cell invasion has been and is still considered helpful in order to take advantage of the progress made in those fields. Concerning the mechanisms involved, it must be emphasized that trophoblast and uterine luminal epithelium (ULE) do not undergo a complete switch to a mesenchymal programme (do not undergo a complete EMT) but make use of partial changes in the epithelial programme. The large number of data accumulated recently should allow us to now make progress in identifying what these partial programme changes are exactly and how they are regulated; also, they may offer chances for obtaining deeper insights into the regulation of implantation.

## 1. Introduction

**Figure 1:**
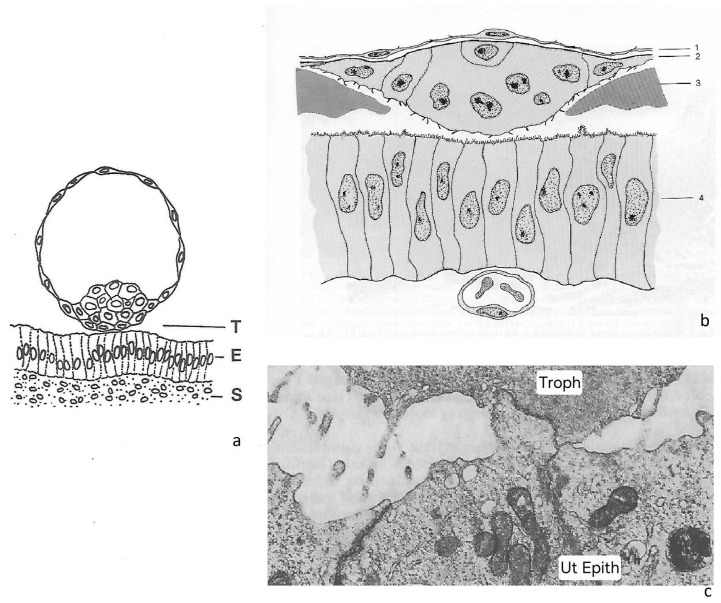
The initial phase of embryo implantation: two epithelial tissues, the trophoblast of the blastocyst and the epithelium of the endometrium (ULE), initiate an adhesive interaction with their apical cell poles. This presents a cell biological paradox because apical cell membranes of simple epithelia are normally non-adhesive but repellent. In case of embryo implantation, a complex sequence of cell–cell interactions then follows, in most species accompanied by invasion of the trophoblast through the uterine epithelial lining. (**a**) The topographical situation as found in the human (T = trophoblast; E = epithelium of the endometrium; ULE; S = endometrial stroma) (from [17,18] with permission). (**b**) Rabbit (1 = abembryonic endoderm; 2 = trophoblast; 3 = blastocyst coats, zona equivalents; 4 = ULE) (from [19] with permission). (**c**) The trophoblast (Troph) adheres to part of the apical plasma membrane of a uterine epithelial cell (Ut Epith) in the ferret. Adhesion appears to be so firm that part of the apical cytoplasm of the uterine epithelium is drawn towards the trophoblast (from [5,20] with permission).

Embryo implantation in the uterus is an astonishing, even perplexing process when looked at from a cell biological point of view. It starts with the formation of cellular contacts between the blastocyst and the endometrium via their epithelial cell linings, the trophoblast and the uterine epithelium (Figure 1a–c). This is true for all species, regardless of their type of implantation: invasive (haemochorial: e.g., human; endotheliochorial: carnivores), moderately invasive (ruminants), or non-invasive (epitheliochorial: pig) [1,2,3,4]. However, cell biology tells us that such a start is far from trivial: both partners, the blastocyst as well as the endometrium, are covered by simple epithelia, and their apical plasma membranes (i.e., the region of first contact) would be expected to resist contact formation, if behaving in the same way as other, orthodox epithelia do in many organs. It is now about 40 years ago that this puzzling fact was identified as a research topic and started to receive attention as a “cell biological paradox” [5,6,7]. Understanding this unusual cell behaviour at implantation initiation became a major research goal, raising the hope that this focus might open new experimental and therapeutic approaches.

The recent literature indicates that in the meantime it has become quite commonplace to regard implantation through such a cell biological lens. Topics which are receiving particular attention are epithelial cell properties like membrane composition [8,9], cell polarity [10], and the concept of epithelial–mesenchymal transition (EMT, i.e., the concept that epithelial cells can switch their structural and functional programme to adopt an organization more typical for mesenchymal (connective tissue-type) cells which would enable invasion (for an actual definition of EMT see [11])). Recently, implantation has not been infrequently addressed bluntly as an EMT event [12,13,14,15,16]. However, the basic reasons why the attention of implantation researchers was originally directed towards polarity aspects as well as to the EMT concept [5,6,7] are rarely reconsidered today. Are there really no open questions about basing implantation research on this theoretical framework, or about any limitations that such an approach may have? Has research based on these concepts allowed us already to gain sufficient insights into implantation failures?

Implantation rates in assisted reproduction (in vitro fertilization—embryo transfer, IVF-ET) are still not quite satisfactory, in spite of much research. Since blastocyst quality assessments performed to date do not really improve the situation, endometrial factors remain a major aspect that needs to be considered [10,21,22] (for additional literature see [23] along with other contributions to the present Special Issue of *Biomolecules* [24]). The apparent complexity of blastocyst–endometrial interactions at implantation is assumed to be a factor behind the high early embryo mortality in human reproduction [25]. Thus, we could ask whether the particular cell biological focus that started about 40 years ago, i.e., on regulations acting through changes in basic epithelial characteristics of the uterine lining and of the trophoblast [5,6,7], was at all helpful for gaining a significantly better understanding of the mechanisms and perhaps for improving therapy. Might there be a reason to also ask whether all aspects of this specific research concept have indeed been sufficiently exploited so far?

The actual state of implantation research is discussed in a number of other contributions to this Special Issue of *Biomolecules* [24]. In the present introductory assay, a look is taken at the history of thought and concept development, why and how the focus was redirected to the role that epithelial cell polarity and EMT-like processes play in implantation, at the advances made, and at future research directions. Also, it will be asked how fitting these theoretical concepts should be seen now in the light of published findings. For example, in the original literature on using the EMT concept for explaining endometrial receptivity and trophoblast invasion, it was emphasized that these cells do *not* undergo a complete EMT (epithelial cells do not really take up an entirely mesenchymal phenotype, but they rather make use of only certain parts of such a programme change) [5,6,7]. This is clearly not reflected in many of the more recent studies on implantation, although some authors do now emphasize this aspect [16,26]. Does this concept remain important for implantation research? In tumour invasion research, “partial EMT” has recently been recognized as a major topic for discussion [27,28]. In some areas like wound healing, it has been argued that it might be more appropriate to use a term like “epithelial–mesenchymal plasticity (EMP)” instead [29]. In contrast, some authors point out that changes in the expression of certain individual cell parameters may be sufficient for inducing a switch in cell behaviour of tumour cells to developing invasive growth, e.g., changes in adhesion molecule expression. It was argued, therefore, that concepts focusing on a more global reprogramming would be dispensable [30]. Also, a general criticism concerning the term EMT can be found in the recent literature, arguing that EMT may be a misleading designation since tumour cells, instead of gaining a full arsenal of mesenchymal markers, rather switch to expressing, e.g., some neural markers such as N-cadherin [31]. This might in the first place be a question of terminological accuracy, as it does not necessarily undermine the main ideas behind the EMT concept (or whatever new term might be most appropriate to designate it). When taking such controversial views together and asking where we stand in implantation physiology, it may thus be of value to reconsider the main cell biological facts which had originally led to proposing an application of cell biological concepts, including the EMT theory, to implantation research, and to confront this with recent data. This will be the main intention of the present text.

## 2. Reconsidering Major Facts That Directed Implantation Research Focus on Epithelial Cell Polarity and the EMT Concept

At the time when the epithelial cell polarity paradox of implantation was identified as a conundrum and as a promising field for implantation research [5,6], cell biology had just developed a new focus on general cell organization and its combination with cell behaviour, and, in particular, on apico-basal polarity of epithelial cells and their structural and molecular characteristics, as well as on regulatory processes involved in polarity maintenance [32,33]. It was also at that time that the concept of epithelial–mesenchymal transition (EMT) was developed [34,35,36], a theoretical framework emphasizing changes in cell organization and behaviour in development, in which cell polarity plays a major role. Embryo implantation was originally not included in the theory by Elisabeth Hay. Thus, exploring its application to implantation research appeared appealing [6,7]. Consequently, many research projects using these new avenues of thought were launched (main topics already covered during the first 20 years are listed in [37]). In the following sections, I will reconsider the major facts on which the refocusing of implantation research on epithelial polarity and the EMT concept were based.

### 2.1. Choice of Focus on Endometrium vs. Trophoblast

#### 2.1.1. Trophoblast

As morphology suggests, the trophoblast of blastocysts takes an active part in implantation, including adhesion to and (in species with invasive implantation modes) invasion into endometrium. Initially, there are only certain parts, and not the whole circumference, of the blastocyst trophoblast cell population which mediate the adhesion to the uterine epithelium (e.g., in the human and the rhesus monkey: trophoblast of the embryonic pole of the blastocyst [38,39]; in the rabbit: initially abembryonic trophoblastic knobs, or in carnivores like the western spotted skunk or the ferret, trophoblast plaques [19,40,41,42,43], Figure 1b,c). These parts of trophoblast acquire apical membrane adhesion competence which indicates that elements of their apico-basal polarity must have undergone changes. This is not a trivial process, however, since the trophoblast is defined as an epithelial tissue [44]. Somewhat similar polarity changes are observed in the partnering tissue, the uterine epithelium, when it enters the state of receptivity (the endometrium will be discussed more in detail below). It was proposed that all these cellular changes may perhaps involve parts of an EMT programme [5,6,7]. In most species specialized trophoblast cells subsequently express invasive behaviour, to degrees that vary depending on the type of placentation, while in the pig, the trophoblast just attaches to the uterine epithelium and does not exhibit invasive behaviour in the uterus (epitheliochorial placentation); in ruminants (synepitheliochorial/syndesmochorial placentation) trophoblast cells intercalate and fuse with uterine epithelial cells, thus just keeping purely epithelial-type interactions, although now with another type of epithelial cell. However, in these species they do not penetrate into the endometrial stroma. In endotheliochorial (carnivores) and haemochorial (human, rabbit) placentation, in contrast, they subsequently do develop the ability to interact with stromal tissue, which requires a different set of adhesive properties and of lytic enzymes, enabling them to invade more deeply [1,2,3,4,45,46,47,48,49].

The assumption that adhesive interactions between trophoblast and the uterine epithelium are indeed critical for initiation of implantation is not only suggested by morphology, but mechanisms involved can be studied in detail with the aid of various in vitro model systems. Such model systems even permit to obtain data on the degree and time scale of adhesive force development as measured by atomic force spectroscopy [50]. Some of the model systems provide insights into signalling and cell reorganization processes that are involved. I will come back to the model systems and insights they provided later in a special paragraph.

Trophoblast cells develop their adhesive and invasive properties in an autonomous manner, from late blastocyst stage on. However, the mechanisms behind those changes in cell properties and behaviour, any regulations, and how the trophoblast differentiation clock is timed still largely remain a mystery [21]. In particular, it remains unclear what regulations may determine these changes in the late blastocyst when trophoblast cells first gain their apically adhesive (and invasive) properties [51]. This does not seem to depend in any way on instructive regulations from the maternal/uterine side. In vitro, the trophoblast of blastocysts develops the ability to adhere even in the absence of endometrial tissue or any specific endometrium-derived matrix (embryo outgrowth model [52,53,54]). In vivo, mouse trophoblast has been shown to invade deeply regardless of the hormonal status of the host if transplanted to ectopic sites (e.g., under the kidney capsule), even in males [55,56,57,58]. In the pig, a species with epitheliochorial implantation where the trophoblast does not penetrate the uterine epithelium in vivo [1,48,59,60], blastocyst trophoblast was reported to nevertheless be able to attach to fibroblasts in vitro [61], and even to exhibit a degree of invasive behaviour when transplanted to ectopic sites [62]. It should be noted, however, that in these experimental situations the trophoblast does not have to interact with the apical cell pole of an epithelium. Trophoblast cell surface molecules that are involved specifically in attachment to uterine epithelium have more recently been studied intensely in species with the central implantation mode (large farm animals, e.g., ruminants), and results indicated that aspects of EMT-like cellular changes seem to be included [14,46,63].

In the human, trophoblast of placentation stages (i.e., stages later than the blastocyst stage, e.g., anchoring villi) has often been used in model studies and has revealed many data on the role of the state of differentiation, as well as of adhesion and invasion-related parameters [45,47,64,65]. Application of the EMT concept to the anchoring villi model was already discussed at a relatively early time point [7,66]. Two- and three-dimensional models employing human choriocarcinoma cell lines, or trophoblast isolated from placentae, have been used in order to study effects of modulating the state of differentiation of the trophoblast in vitro and provided insights into its relevance for attachment, either to matrix or to uterine epithelial cells, and for the general invasive potential of the trophoblast [64,65,67]. However, the most critical parameters which may mediate the potential for trophoblast adhesion and invasion into endometrium via the uterine epithelium remain to be identified—in a three-dimensional in vitro model using several choriocarcinoma cell lines and human endometrium, those trophoblast cell lines which were most aggressive in a general invasion assay (not involving uterine epithelium) were not the same as those which most efficiently adhered to and invaded into human endometrium via the epithelial route. This clearly demonstrates that specificities of epithelial interactions play an important role for this selectivity, in contrast to a lesser degree of selectivity seen in stromal invasion [68,69].

Recent research utilizes transcriptomics and proteomics of trophoblast cell populations for studying the differentiation of trophoblast stem cells and placentation, including aspects of EMT-like changes and key signalling pathways [70,71,72,73,74,75]. Increasingly, three-dimensional trophoblast models of various kinds (often called organoids) are used [76]. However, with regard to the initiation of the implantation cascade, it is not clear to what extent such models may sufficiently mimic properties of blastocyst stage trophoblast [77]. Regulatory principles behind the gain of implantation competence by blastocysts [21] still remain largely elusive.

Stem cell-derived human trophoblast (e.g., being a constituent of blastoids) has been proposed as a promising new model tool in this regard [77,78,79], but information on cell–cell interactions obtained with this type of model remains limited [80]. However, the production and use of human embryoids (including blastoids) raises ethical concerns [79,81]. Warnings over many years have expressed concern regarding human stem cell embryo models which may possess complete developmental potential; non-human (primate) models should be used instead [82,83,84] in spite of the fact that human-specific molecular specificities exist [85]. The newest modifications of the ISSCR Guidelines move in this direction, proposing policy change to greater restrictions for the use of those human stem cell-based embryoid models (SCBEMs) which possess a high degree of potentiality for complex development [86], a move that was certainly warranted.

In summary, it may be concluded that studies using various trophoblast models, although of much research interest, have so far not really provided a handle on how attachment and invasiveness of late blastocysts are regulated, and how implantation competence of blastocysts can possibly be modulated experimentally and may perhaps be adjusted therapeutically, e.g., in IVF-ET (in vitro fertilization—embryo transfer) contexts [21]. Recent studies on the regulatory mechanisms behind dormancy-like cell stages may in the future provide better insights into some of the cellular changes behind the gain of implantation competence of blastocyst trophoblast [87].

Apart from mediating adhesion to uterine epithelium and invasion, blastocyst trophoblast is involved in signalling to the maternal system. In the present article I will not discuss the systemic effects of blastocyst/trophoblast signalling to the maternal endocrine system via, e.g., chorionic gonadotrophin (hCG) secretion in the human, and interferon-tau (IFN-tau) in ruminants [1,49,88]. I will further below address aspects of local signalling of the blastocyst to the endometrium insofar as it relates to local changes in cell organization seen in the adjacent uterine epithelium.

In the following text I will focus on the endometrium and specifically on the uterine luminal epithelium.

#### 2.1.2. Endometrium

The endometrium, and, in particular, its epithelium lining the cavum (luminal epithelium, addressed in the following sections as *uterine luminal epithelium*, *ULE*), is of particular interest since there is good evidence that it has an important regulatory role in implantation, under maternal hormonal control. The changing physiological states of the ULE that are controlled by steroid hormones (directly or indirectly via interactions with the endometrial stroma) seem to be decisive for “receptivity” or “non-receptivity” for attachment and invasion of the blastocyst trophoblast, defining an “implantation window” ([89]; for more recent literature, see [23,90,91,92,93] and other contributions to this Special Issue of *Biomolecules* [24]). It has been shown that mouse blastocysts can initiate implantation in the uterus completely independent of any hormonal control if the epithelium is removed [94]. This demonstrates that outside the implantation window, the ULE acts as a barrier, but this barrier becomes somehow surmountable at receptivity, permitting trophoblast cells to initiate a cascade of adhesive and (in many species) invasive interactions. The complexity of the responses of the endometrium to signalling and of its interactions with the blastocyst may also be critical for embryo selection mechanisms (i.e., selection of “healthy embryos”) [95]. The hormonal control of endometrial cell behaviour offers very attractive handles for experimental approaches. In addition, hormonal regulation of endometrial states is of interest for clinical applications.

The ULE seems to be a unique type of epithelium showing properties that are not matched by any other epithelium in the adult body [96], i.e., the ability to allow and control implantation in vivo, by undergoing structural and functional changes that are crucial for the different physiological states of “receptivity” and “non-receptivity” of the whole endometrium. Other epithelia in the body are not known to be able to develop a state at which they would permit trophoblast attachment and invasion, including the tubal epithelium through which the trophoblast cannot penetrate in any hormonal state, at least not in animals [97,98] (this is often debated for human tubal epithelium due to the occurrence of tubal pregnancy; however, epithelial defects may suffice there). Interestingly, receptivity of the uterine epithelium even seems to show selectivity for only certain types of adhesive cells (i.e., for trophoblast), not allowing other types of invasive (e.g., various types of tumour) cells to adhere and invade from its apical side, and receptivity also seems to show species-specificity [68,69,99].

In addition to acting as a barrier against trophoblast invasion in the non-receptive state, while permitting interaction at receptivity, the ULE serves as a signal transducer between the embryonic and the maternal system, which includes the mediation of signals initiating decidualization of the endometrial stroma in rodents [90,100]. I will focus here on the ULE but will omit a discussion of the manifold mutual interactions between the ULE and the endometrial stroma. Such epithelio-stromal interactions are known to be significant factors in the physiology of all mucosal tissues [101] including the endometrium, although they are definitely specialized in the latter case. These interactions are involved in the hormonal regulation of endometrial functions (for an overview of the literature, see [26,90]) and would deserve a separate chapter, but will not be discussed in the present paper. Aspects of local signalling of the blastocyst to the uterine epithelium will be addressed further below.

## 3. Lessons from History: How Epithelial Polarity and EMT Became a Focus in Implantation Research, Learning from General Cell Biology, Epithelial Fusion Processes in Development, and Tumour Research

Redirecting the focus of implantation research on epithelial cell properties and EMT was far from trivial in about the 1980s, and it may be of value to take a look back at the reasons why this change in interest started at that time point. Until then, research on endometrial physiology had been dominated entirely by studies on endocrine regulations, but not on details of states of cell architecture. This was, no doubt, due to the discovery of estrogens and progesterone and their actions in the 1930s, followed by the creation of their synthetic derivatives, and then by the development of oral contraception. When in about the 1960s cell biology started to boom as an independent field of experimental research, proposals to apply the new results and concepts to implantation research were at first met with reluctance in the reproductive sciences community, a fact that may today appear surprising and difficult to understand. As I remember very well, for example, one of the most prominent researchers in the implantation field (rat and mouse model) at that time, Stanley R. Glasser, complained bitterly in the 1980s that he experienced a complete lack of interest when he started to confront reproductive biology conference audiences with aspects of epithelial cell polarity, regarding interactions of trophoblast and endometrium at implantation. He, like others, had previously concentrated on the regulation of uterine gene expression by estrogens and progesterone, specifically in relation to decidualization [102]. The new focus on epithelial cell polarity was first discussed by them with regard to certain in vitro models of implantation [103,104,105,106]. The main message was that uterine epithelial and trophoblast in vitro models should be designed in a way to ensure they would express an appropriate cell polarity, in order to provide meaningful insights.

### 3.1. Early Approaches: On the Role of Glycoprotein Barriers and Proteinases

Experience has shown how great hurdles were. In the earlier years, the primary focus of most research (including my own) had been different, i.e., on biochemical factors like certain glycoproteins and enzymes and their role in implantation. Cell surface-bound glycoconjugates (glycoproteins, glycolipids) present on the ULE and the trophoblast as an apical glycocalyx were recognized to be potential mediators or barriers for attachment and implantation (in particular sialomucins, sulfomucins) [107,108,109,110]. In addition to cell surfaces, this research included the constituents of the zona pellucida and the more complex blastocyst coat structures found in many species [111] (for later developments in research on the role of such glycoconjugates in regulating adhesive interactions, e.g., MUC1, see [8,112]). It is surprising how long it took until the barrier role of negatively charged glycoconjugates/glycoproteins, in particular certain sialomucins (like podocalyxin, episialin), received the broader attention of experimentors. This focus is now experiencing a renaissance in the context of functional tests for studying trophoblast adhesion [113,114,115].

The focus of interest then switched as it was found that certain specific *proteinases* (e.g., *blastolemmase*) played a crucial role in the initial phase of implantation in the rabbit, i.e., in hatching from the blastocyst coverings (coats, like zona pellucida, mucoprotein coat, and neozona). The hatching process is a prerequisite for implantation initiation and it was indeed possible to block implantation very efficiently when appropriately selected proteinase inhibitors were administered into the uterine cavity in vivo [19]. When certain non-toxic and well-tolerated inhibitors were administered (e.g., aprotinin), their blocking effect (in the rabbit) was very specific for the process of hatching from the extracellular coverings (coats, zona equivalents), the blastocysts remained alive as long as such intrauterine treatment was continued (using an osmotic minipump), and those of them which did not hatch mechanically but stayed encased in their non-dissolved coverings even continued their development to quite advanced post-gastrulation and neurulation stages while remaining unattached and non-implanted in utero, in the rabbit [116,117,118]. Some of the blastocysts managed to hatch mechanically so that the barrier (the coverings) did not intervene anymore, and in that case their trophoblast showed a late and locally restricted attachment to and fusion with the uterine epithelium.

The conclusion was that the investigated serine proteinase system [19,116,118,119] is:

- *not* involved in post-hatching processes as had originally been expected, i.e., not in contact formation between trophoblast and uterine epithelium (in contrast to previous studies which had led investigators to suggest that secreted uterine proteinases act as an “implantation initiating factor” [120,121]),
- *not* involved in invasion through the ULE as many had expected to be the case (however, there is evidence for a role of metalloproteinases in stromal invasion; see [122,123]),
- *nor* does implantation have any morphogenetic function (like directing body axis development, etc., as was assumed by other authors using the mouse model) (discussed in [124]).

Therefore, it appeared necessary to look for alternative explanatory concepts in order to understand the cellular events at implantation initiation. This initiated the search for a new theoretical approach.

### 3.2. The Switch to Studying Epithelial Cell Polarity Aspects

In the 1980s, general aspects of the *structural and functional organization of epithelial cells*, and, in particular, their *polarity*, had become a new focus of cell biology [32,33,125]. Why should not these new views be applied to implantation research? At that time, however, it still appeared quite audacious to confront the implantation research community with these new concepts of general epithelial cell biology. It may appear strange today that in a review from 1983 I still felt I had to use my words very cautiously when concluding (after referring to the limited number of electron microscopical and histochemical findings that we had at that time point): “*Does all this indicate that, perhaps, the uterine epithelium loses or even reverses its functional (apical-basal) polarity, after proper hormonal conditioning, when the trophoblast contacts it? This might be one factor contributing to the introduction of changes in the properties of the apical cell surface of the uterine epithelium, … Does this apply to the attachment between trophoblast and uterine epithelium?*” ([126], p. 34). As far as I can recall, this may have been the first time in the literature that anyone addressed the cell biology of implantation initiation in this pointed way, focusing on cell polarity phenomena and their consequences for epithelial cell behaviour concerning attachment and invasion.

In order to trigger interest of the research community, it appeared helpful to address implantation as a “*cell biological paradox*” [5,6,7]. This was based on the following main facts and logic:

(i). Both the trophoblast of the blastocyst and the uterine epithelium are typical mono-layered (“simple”) epithelia.
(ii). Such epithelial cells normally invest a complicated intracellular machinery to maintain a polarized architecture which includes an apical membrane with non-adhesive properties.
(iii). Nevertheless, the blastocyst attaches to the receptive uterine epithelium exactly via this apical surface that regularly ought to be non-adhesive.
(iv). Studying cell biological details behind this change in behaviour of the uterine epithelium might provide insights into the regulation of endometrial receptivity for trophoblast adhesion and invasion and into the mechanisms of hormonal control of implantation.

### 3.3. An Examination of Epithelial–Epithelial Interactions as Observed in Developmental Fusion Processes (EFPs)

**Figure 2:**
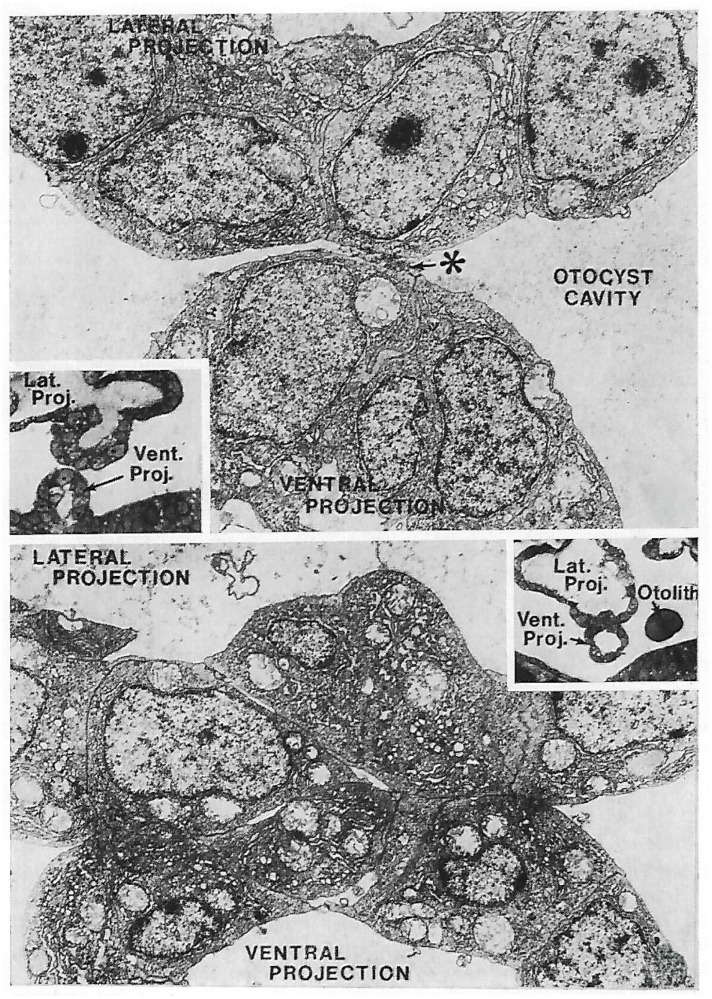
An example for an epithelial fusion process (EFP) during development of the inner ear, here during the formation of the semicircular canals (in Brachydanio rerio). Two projections of the ear vesicle move towards each other, their apical membranes start to adhere (some extracellular material, indicated by the asterisk, may be involved in mediating this), and it comes to a rearrangement of the cells while the original apico-basal orientation changes, so that finally a communication will be formed between the mesenchymal spaces (above and below) Lat. Proj. = lateral projection; Vent. Proj. = ventral projection. (from [5,147] with permission).

**Figure 3:**
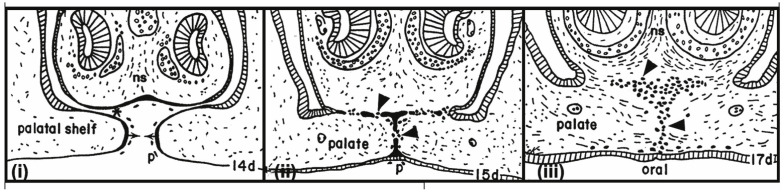
The fusion of palatal shelves during secondary palate development, which starts with contact formation between two epithelia, has served as a basis for formulating the EMT concept [159]. Shown are three consecutive developmental stages, (**i**) = 14 days, (**ii**) = 15 days, (**iii**) = 17 days. ns = nasal septum. The palatal shelves fuse together and with the nasal septum by transforming the adherent epithelial seams to mesenchyme to become confluent. The dark cells (arrowheads) show diagrammatically the relative contribution to mesenchyme made by these epithelial seam cells. (from [180] with permission).

Looking at comparable processes of epithelial–epithelial interactions anywhere in developmental biology was interesting. Indeed, a number of examples exist in embryology where certain morphogenetic processes start by interactions between two epithelia via their apical ends, i.e., the so-called *embryonic fusion processes* (EFPs) (e.g., in inner ear development, fusion of nasal processes and palatal shelves, neural tube closure, as well as during septation of the heart where endothelial cells are the interacting partners) (Table 1; [5], Figure 2 and Figure 3). In the course of these very divergent developmental processes, opposing epithelia surprisingly establish a first cellular contact via their apical plasma membranes. In combination with this process, cells change many parameters of polarization and behaviour, and subsequently the cells of the two attached epithelial linings move apart and give way, so that the connective tissue spaces of the fusing partner structures can now join. The modified epithelial cells even become invasive in some of these “models” (e.g., the neural crest cells at neural tube closure, and endocardial cushion cells at septation of the heart) and they may acquire certain mesenchymal-type properties and may merge with stromal cells.

Remarkably, a number of changes in epithelial cell architecture, molecular composition, and behaviour were found to be common to all these systems, starting before attachment and continuing during the fusion event, and were comparable to phenomena seen in implantation [5]:

- At the apical cell pole: changes in glycocalyx composition, smoothening of the profile of the plasma membrane, loss of microvilli; in some cases, also the formation of peculiar apical cell processes.
- At the lateral plasma membrane: changes in the distribution of junctional complexes.
- Basal cell pole: changed integrity or complete loss of basement membranes (in some cases starting already before attachment of the fusing partners).
- Dissociation of some of these epithelial cells and acquisition of migratory motility as individual cells within extracellular matrix (i.e., invasive behaviour).
- In some of these EFPs, regulation by various growth factors and steroids was found (in case of palatal shelves: glucocorticoids).
- Although cell death was observed, no evidence was found for any major mechanistic role that it would play here (although in the case of palatal shelves this was disputed by some authors).

Since all of the observed cell changes are features connected with apico-basal polarity of epithelial cells, it was concluded that in all these examples of developmental processes (Table 1), a destabilization of the apico-basal polarity of the interacting epithelial cells occurs; this may be a precondition and a starting point for the changed cellular behaviour (adhesion via apical cell poles and subsequent invasion) [5,6,7]. The term “destabilization” was used on purpose when now comparing with the special situation of trophoblast–endometrial interactions at implantation, considering the role of local signalling between the implanting blastocyst and the endometrium which may lead to completion of the structural and functional changes in the ULE at the attachment/penetration site.

The general idea behind comparing implantation to entirely different developmental processes was to find common traits, an approach which may widen the scope and enable new mechanistic and molecular insights into endometrial receptivity [5,96]. This idea of “looking over the fence” may still have its value since application of an interdisciplinary approach can take advantage of research progress made in studying other developmental processes (a recent example may be genes involved in EFPs, like GRHL2 interacting with noggin [181], or the recently renewed interest in the role of TGF beta 1 and 3 in secondary palatal shelve fusion [166]).

### 3.4. Impulses from Taking a Side-Look at Tumour Invasion and Metastasis

Surprisingly, when proposing to apply such a generalizing cell biological view to implantation research, we initially experienced much scepticism, in particular when trying to compare with tumour research. It had appeared very reasonable to us to try learning from tumour research, particularly since in vitro studies with various highly invasive tumour cells had revealed that in those models, tumour cells did not invade via the apical cell pole of host epithelia, although they did invade into exposed connective tissues, and that initial adhesion processes between tumour and host were critical [182]. In 1986, my group organized the annual meeting of an international tumour researchers association (Cell, Tissue, and Organ Culture Study Group, C.T.O.C.) and we invited a number of international implantation researchers to discuss aspects of cell–cell interactions that may be common in all these systems, specifically comparing research on tumour invasion with embryo implantation [183]. To my knowledge this was the first time that epithelial cell polarity was discussed at a conference that focused on embryo implantation (the implantation-related part of the proceedings was published years later [184]). Today it may appear surprising that it turned out quite difficult to obtain financial support for such a conjoint conference. One reviewer expressed that he felt it would not make sense for an implantation researcher’s group to organize a tumour researchers’ meeting, and he thus voted for denying financial support. A rebuttal proved difficult but was finally successful.

**Figure 4:**
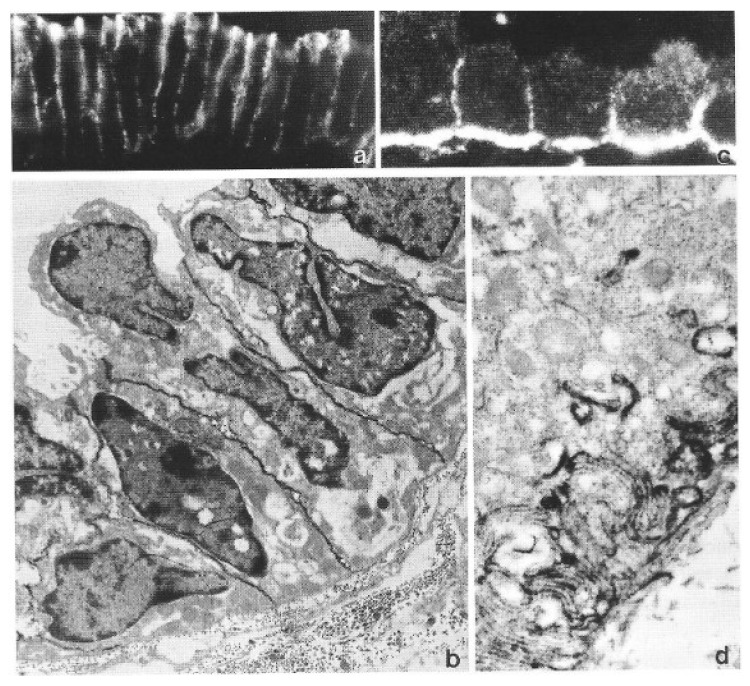
Changes in apico-basal polarity of the uterine epithelium at receptivity, as indicated by a redistribution of an adhesion protein (E-cadherin) at the lateral plasma membrane. (**a**) In the non-receptive (non-pregnant) state, rabbit ULE shows a distribution of E-cadherin on the lateral membrane as typical for polarized simple epithelia, i.e., maximal concentration in the subapical junctional belt region while less is found in the other parts of the lateral plasma membrane, and none in the apical and basal domains. Light microscopical immunohistochemistry, FITC labelling, 680×. (**b**) The subapical maximum is largely lost at receptivity. Intermediate part of an endometrial crypt in the paraplacental region, 8 days p.c. TEM, horseradish peroxidase labelling, pre-embedding immunohistochemistry on a thick cryosection of PLP-fixed material, 3500×. (**c**) Certain subpopulations of oligonuclear ULE cells show extreme degrees of E-cadherin redistribution in endometrium of rabbit paraplacental fold, 9 days p.c. Here, the maximal concentration of this adhesion protein is found in a very unusual location, i.e., in the basal membrane region (below). The apical plasma membrane (above) remains free of E-cadherin. Light microscopical immunohistochemistry, FITC-labelling, 510×. (**d**) Electron microscopically, the basal plasma membrane region of this type of modified ULE cells, shows that E-cadherin is highly concentrated at the membranes of basal projections of these cells and seems to play a role in contact formation of these projections with each other. The stromal space is at the lower righthand corner. Labelling as in (**b**) (from [219] with permission).

**Figure 5:**
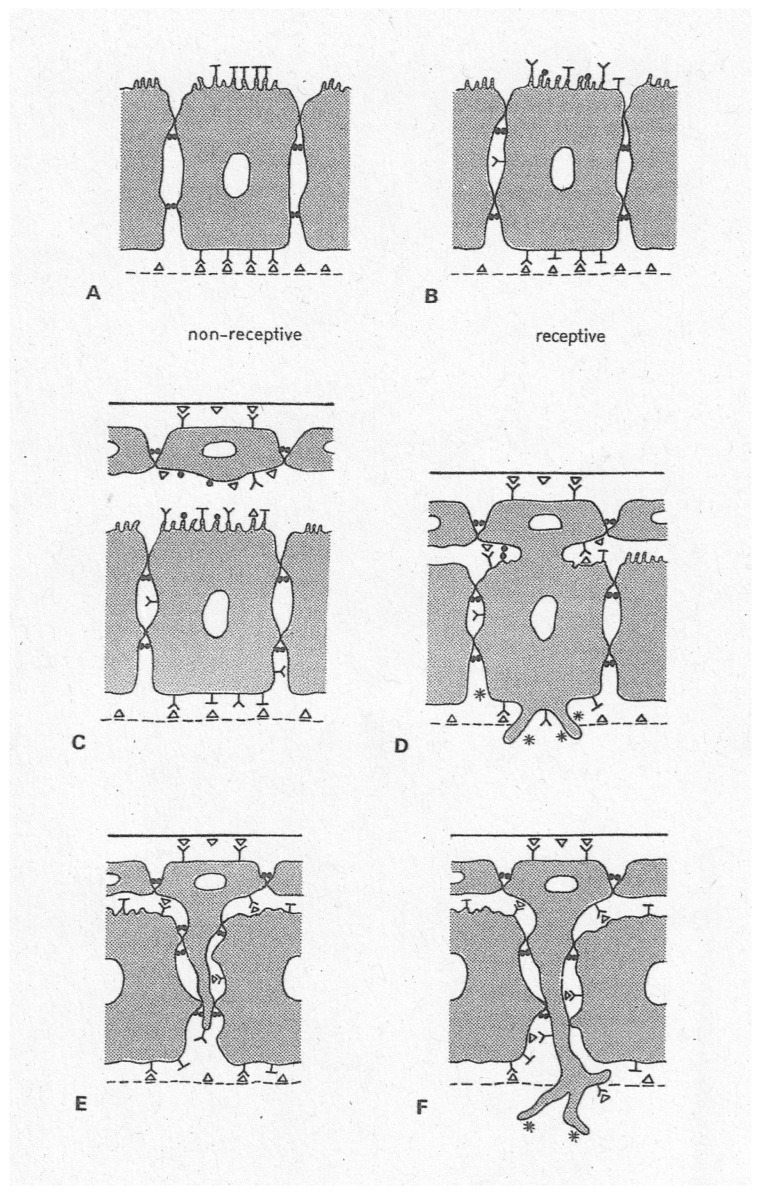
Historical sketch presenting original concepts about changes in cell polarity of trophoblast and uterine epithelium at implantation initiation, focusing on redistribution of adhesion molecules. Whereas the uterine epithelium (ULE) expresses non-adhesive properties (molecules) at its apical plasma membranes during the preimplantation phase (non-receptive state, (**A**)), there it expresses adhesion receptors when acquiring receptivity (**B**). The latter also applies to the invasive trophoblast cells (above, (**C**)). Penetration of trophoblast (above) through ULE (below) occurs by fusion (rabbit, probably human; (**C**,**D**)) or intrusion (carnivores; (**E**,**F**)). Symbols: T, apical-type, repellent molecules (glycocalyx); solid circles, cell–cell adhesion molecules; Y, heterotypically binding receptors; triangles, ligands for Y receptors; asterisks, matrix receptors like integrins. This sketch was not meant to be correct in molecular details but intends to illustrate the cell biological principles (from [6] with permission).

As the proceedings of that conference [184] show, many data available already at that time documented changes in epithelial cell characteristics, in particular in cell polarity-related parameters, associated with endometrial receptivity and trophoblast invasiveness, which served as a starting point for a novel look at implantation ([6], Figure 4 and Figure 5; Table 2). In the receptive ULE, quite a number of parameters related to epithelial properties deviated from the orthodox epithelial catalogue, in particular characteristics of apico-basal polarity. Changes in cell organization were found not just at the apical cell pole but throughout the whole cell. Polar organization was well expressed in the ULE during the pre-receptive phase, as typical for any simple epithelium, but at receptivity and, in particular, in the implantation chamber, quite a number of polarity features were down-regulated and some of them even inversed (Table 2, Figure 4 and Figure 5). For example, the apical plasma membrane lost a number of typical marker enzymes as well as components of the glycocalyx. In contrast, this membrane gained the potential to form junctions that are normally found only at lateral or basal plasma membranes, like gap junctions and hemidesmosomes. At the lateral plasma membrane, the typical polar (subapical) distribution of various junctions was found to change to a more even distribution (for a review including more recent findings along these lines see [23]). At the basal cell pole, basement membrane production and adhesion to it become defective. In summary, considerable loss of features of apico-basal polarity was found in the ULE at receptivity. More general epithelial characteristics were also found to change, concerning the cytoskeleton (vimentin up-regulation) as well as intracellular transport systems. The lipid composition of ULE membranes also changed [185], which today receives renewed interest [186]. This led to the conclusion that receptivity is accompanied by quite global changes in the organization of the uterine epithelium, not just apical membrane changes, causing it to deviate from the expression of many of the properties of an orthodox simple epithelium. It was concluded that the ULE down-regulates a number of its typical epithelial properties when it attains receptivity and during penetration by the trophoblast [5,6].

**Table 2:** Endometrial receptivity: observed changes in epithelial cell characteristics from which the hypothesis of partial EMT originated (data from [5,6,7] with selected recent references added).

Observed Changes	References
**STRUCTURAL ORGANIZATION** ***Plasma membranes:*** ***Apical plasma membrane:*** *Loss of apical marker molecules:*	
Loss of brush border-type enzymes	[7,187,188]
Loss of glycocalyx components and changes in glycocalyx composition (including lectin-binding patterns) (for the more recent literature see [8,189])	[190,191,192,193,194,195,196]
*Gain of basolateral membrane characteristics:* Increase in intramembranous protein particle density to equal basolateral membrane	[197,198]
Gain of receptors for matrix/cell surface molecules	[46,196,199]
Gain of the ability to form hemidesmosome-like junctions	[19]
Gain of the ability to form “reflexive” gap junctions	[200]
***Lateral plasma membrane:*** Loss of polar (subapical) maximum of desmosomal proteins (desmoplakin), more even distribution gained	[7,23,96,188]
Reorganization of tight junctions, loss of polar (subapical) concentration, and proliferation of strands basally	[201,202,203,204]
Changes in organization of adherens junctions, loss of polar (subapical) maximum of E-cadherin, and more even distribution (Figure 4)	[7,204,205]
Gain of integrin alpha6, redistribution from basal membrane domain	[206]
***Basal plasma membrane and basement membrane:*** E-cadherin expression in extreme cases	(Figure 4c,d)
Reduced adhesion to basal lamina	[207,208,209]
Defective basement membrane	[210,211]
***Cytoskeleton***	
Vimentin upregulation and intracellular redistribution	[205,212]
**FUNCTIONAL CHARACTERISTICS AND CELL BEHAVIOUR** ***Intracellular/transcellular transport***	
Endocytosis and transcellular transport changes	[213,214,215,216,217,218]
***Cell behaviour*** Homotypic cell–cell interaction maintained (epitheliochorial placentation: pig) or weakened (invasive types of implantation: rodents, human, and carnivores) Potential for heterotypic cell–cell interaction with trophoblast gained (all mammals)	
Adhesive interaction with basement membrane (BM) weakened: sloughing from BM in rodents	[207,208,209]
Penetration of uterine epithelial processes through BM: rabbit, human	[210,211]

### 3.5. Endometrial Receptivity Compared with the EMT Concept

A further stimulus for expanding implantation research along these lines was that Elizabeth D. Hay developed her theory about epithelial–mesenchymal transitions (EMTs) in development [34,35]. This EMT theory refers to some of the EFPs already addressed above (e.g., the formation of the secondary palate), and remarkably, structural and functional cell polarity is addressed as a central point (Table 3). However, the EMT literature originally included neither endometrial receptivity nor trophoblast invasion. On the other hand, when in subsequent years several groups applied the EMT concept to the field of tumour cell invasion, international interest increased considerably. This also motivated us to ask how closely related EMT mechanisms may be to implantation physiology. Various in vitro systems were developed in order to check for functional significance of component processes, which I will address briefly in a separate paragraph further below.

Molecular marker profiling to define and characterize EMT processes has received much attention [225]. However, aspects of structural and functional organization of cells are still main criteria. In the recent Consensus Statement on the definition of EMT processes it is stated that “*The primary criteria for defining EMT status should be changes in cellular properties together with a set of molecular markers, rather than relying solely on molecular markers*” ([11], p. 349). This is much in agreement with the original approach used when comparing findings on endometrial receptivity and trophoblast invasiveness with those on which the EMT concept was based (and, in particular, with the developmental EFPs mentioned above) [6,7,96]. It was evident from the start that *cell polarity* aspects stand out in all these cases. Table 2 and Table 3 focus on the main facts that originally prompted this comparison (for an updated definition of EMT and for recent data on molecular mechanisms involved see [11], and for a comparison with more recent data on processes involved in endometrial receptivity see [16,23,26], as well as other contributions to this SI of *Biomolecules* [24]). Down-regulation of epithelial apico-basal polarity is a major aspect of the entire EMT theory, particularly in tumour research [11,226]. In the uterine epithelium, a remarkable number of characteristics of the apico-basal polarity become down-regulated at the receptive state, as noted from the start and as already mentioned above (Table 2, [5,6]). During the pre-receptive phases, many markers are organized in a polarized fashion along the apico-basal axis, but acquisition of receptivity leads to a loss of this polar organization. Impressively, polarity can even become partially inverted in ULE cells, e.g., the capacity for the formation of various types of junctions at the apical cell pole (Table 2). In certain ULE cells of the rabbit implantation chamber, E-cadherin was even found to be atypically expressed at the basal cell pole. The example illustrated in Figure 4c,d is an extreme case, which is not representative for the more modest changes in apico-basal redistribution as seen in most ULE cells during receptivity. However, this example (taken from a subpopulation of ULE cells which are in the process of preparing for symplasmatic transformation) demonstrates changes in apico-basal polarity most impressively [99,219]. For some of the epithelial-type molecules, not only their intracellular distribution but also their general expression is down-regulated at receptivity. This has led to the proposition that steroid hormone action may (directly or indirectly via the endometrial stroma as a result of epithelial–stromal interactions) change the expressed genetic programme of the ULE in such a way that many of the epithelial type differentiation parametres are temporarily down-regulated at receptivity (but not the entire programme) [6,7]. Several of the changes in the ULE are even more expressed in the immediate vicinity of a blastocyst, i.e., in the implantation chamber, indicating a role of local signalling that I will address further below.

## 4. Global Programme Changes vs. Selective Membrane Transformations

Rather than discussing the basis for these relatively global changes in epithelial-type organization, some authors focus on changes in membrane composition of the uterine epithelium (“plasma membrane transformation” [227]; for recent reviews see [8,26]) and on identifying individual parameters that modulate the apical plasma membrane as of particular functional relevance with regard to mechanisms of initiation of trophoblast adhesion to the ULE. As mentioned above, it had been recognized early that carbohydrate components of the glycocalyx deserve special interest, notably the negatively charged sialomucin and sulfomucin (keratosulfate type) compounds, since they are strongly expressed at the ULE as well as at the trophoblast cell surface. These molecules were expected to possess repellent properties for contact formation via the cell surface [108,110,126]. The early histochemical investigations were followed by lectin-binding studies, showing that the uterine epithelium undergoes an indeed impressive modulation of apical glycoconjugate patterns when approaching receptivity [193,228,229,230]. For some of the glycoconjugate moieties (like MUC1), a repellent function for trophoblast adhesion appears probable, while others may serve as adhesion recognition sites [112,196,231] (for an overview, see [8]). Recent studies focus attention on the CD34 family sialomucin podocalyxin [113,114] and these data strongly suggest a repellent function during the pre-receptive phase and down-regulation at receptivity. This was confirmed by functional studies in an in vitro model using Ishikawa cells. However, in vivo experiments have not yet provided compelling evidence for a major functional role, either for adhesion promotion or for a repellent function, for any of the investigated carbohydrate moieties. So, for example, when certain lectins (WGA, S-Con A) were infused into the uterine lumen in order to block glycoconjugate function in vivo in the rabbit model, implantation proceeded undisturbed [232,233]. Somewhat comparable in vivo experiments on intrauterine lectin injections were also performed in the mouse, rat, and hamster [234,235,236], and in these cases an interference with implantation was reported at that time. However, these experiments are difficult to interpret and to extrapolate to other species including the human, not only due to methodological problems but also to the known peculiarity of rodents (mouse and rat) that the receptive endometrium reacts there to intrauterine infusions and to foreign bodies by sloughing of the ULE and by the initiation of decidualization (as known from the deciduoma model). Thus, the mentioned experiments do not prove that the mechanism of action of the lectins was by blocking attachment of the trophoblast to the ULE. This is only one of the aspects that make rodents problematic as models for human implantation. For example, biophysics of implantation are quite different in both systems [237], as is the endocrinology of endometrial reactivity [89,238]. Studying interactions with endometrial stroma, after epithelial penetration [239], may provide interesting insights into events at later but not the initial stages, even in the mouse model, although many differences to the human do exist.

Around the 1980s the described observations about trophoblast–ULE interactions indicated a need for widening the scope to include other aspects beyond glycoconjugate changes at the apical plasma membrane. As discussed above, the observed changes in the receptive uterine epithelium are indeed of a much more global nature and are not restricted to the apical pole. Remarkable changes are found also at the basal cell pole, e.g., leading to detachment from the basal lamina in rodents. Formation of cytoplasmic projections penetrating the basal lamina was observed in the human and the rabbit (Table 2). Moreover, modifications comprise many other aspects of cell organization as already mentioned. Therefore, it appeared appropriate to consider more global changes in programmes determining cell architecture and physiology, perhaps comprising parts of what the EMT concept postulates.

An important aspect is that the ULE cells do not convert to a completely mesenchymal phenotype but rather *maintain a number of epithelial characteristics*. In most species the ULE continues to express the epithelial-type adhesion proteins E-cadherin and desmoplakin (in some species an upregulation of N-cadherin is observed [12,240]). The ULE reorganizes the distribution of its junctions on the lateral membrane, but cells nevertheless maintain their adhesion to neighbouring epithelial cells (Table 2). When in species with invasive implantation trophoblast penetrates between uterine epithelial cells, both partners retain an epithelial character insofar as they still form epithelial cell-type junctions, with their sister cells as well as the invading new partner, the trophoblast. Furthermore, the uterine epithelium does not lose its cytokeratin filaments although it up-regulates vimentin expression [23,205,212,241].

The conclusion is that the uterine epithelium does not completely give up its epithelial nature and does not switch entirely to a mesenchymal phenotype at receptivity. This fact is certainly functionally significant because transformation into an entirely mesenchymal type of cells during receptivity would not be compatible with maintaining essential functions of the ULE as a transport control tissue. During later stages of implantation, this aspect remains relevant at least in the areas outside the immediate blastocyst/trophoblast penetration site(s). If the ULE cells would convert to mesenchymal cells, this would lead to an obliteration of the uterine cavity, since it would cause the opposing parts of endometrium to become joined (like in the embryonic fusion processes, EFPs, discussed above). In species with the central mode of implantation (like pig and ruminants), it is particularly obvious that major epithelial characteristics of the uterine epithelium are maintained at (and after) implantation, in spite of considerable changes in adhesion molecule expression which permit cellular contact with trophoblast (but not with opposing ULE cells), and in spite of changes in transport activities [46,63,88]. It is an important aspect that the completion of the changes in the epithelial characteristics in the ULE remains a local phenomenon, restricted to the immediate neighbourhood of the implanting blastocyst. A somewhat different story is probably the disappearance of the ULE observed in later stages of pregnancy when in the human the decidua capsularis fuses with the decidua parietalis so that the uterine cavity disappears ([4], p. 135). Here, the ULE of two opposing parts of endometrium interact with each other and disappear, without any participation of trophoblast. It is not known whether apoptosis is involved here. That whole process is not yet investigated sufficiently so it remains unknown whether EMT-like processes may be involved in this case.

Under cell behaviour aspects, the original EMT concept emphasizes that the transformation leads to a replacement of the apico-basal polarity by the front-rear end polarity which mesenchymal cells express when they move in extracellular matrix (ECM) ([34,35]; cf. also the recent EMT definitions [11]). In the receptive ULE, however, the acquisition of manifest front-rear end polarity is not observed, and ULE cells do not really become invasive in any species, not even locally where the blastocyst implants, in contrast to (parts of) trophoblast. A sign of semi-invasiveness may be seen in the formation of cellular processes of the ULE at the basal cell membrane, penetrating the basement membrane (Table 2). However, uterine epithelial cells do not dissociate from each other (i.e., do not give up their homotypic cell contacts to neighbouring cells) and do not become migratory as individual, dissociated cells.

Trophoblast cells invade by taking over lateral ULE membrane junctions, while both trophoblast and ULE retain an epithelial-type adhesion molecule repertoire, i.e., they continue interacting adhesively with other epithelial cells. Invasion into stroma as isolated, individual cells is observed in a subpopulation of trophoblast cells in the case of anchoring villi in the human (as mentioned above). However, the mass of trophoblast cells (except for some detaching invasive cells) maintains the lateral contacts (homotypically adhesive) to their neighbours, throughout implantation and placentation, in all species. These cells also maintain the expression of cytokeratins. In conclusion, trophoblast cells also retain many epithelial properties, even in species with invasive trophoblast. This is particularly evident in case of ruminants, where the (semi-invasive) trophoblast elements (which fuse with the ULE) show a (heterotypic) adhesive behaviour with the ULE. Thus, they keep interacting adhesively with another epithelium, but not with the endometrial stroma [49].

Recent studies using genomics, transcriptomics, and proteomics to compare cell programme changes in various EMT processes provide data on signalling pathways that are possibly involved [10,26,93]. In parallel, there is increased interest on the specific connections with apicobasal polarity changes in processes like tumour cell invasion and metastasis [242]. The complexity of regulatory processes behind these transition processes is now recognized and invites studies on intracellular regulatory networks governing cell states in general [243].

## 5. Local Signalling in the Implantation Chamber

Signal exchange between trophoblast and endometrium is an important element in implantation initiation but is still incompletely understood. Many of the phenotypic changes in the receptive ULE are more expressed in the vicinity of a blastocyst than in more remote areas. The earliest local changes in the ULE of the forming implantation chamber include loss of a number of apical membrane marker enzymes. These changes were interpreted as early signs of signalling from the blastocyst to the surrounding endometrium, stimulating a discharge of apical plasma membrane components into the uterine lumen [187,244,245]. Later, it was observed that components of the repellent apical glycocalyx are also highly down-regulated in the immediate vicinity of a blastocyst, but not or to a lesser degree in more remote parts of endometrium (lectin-binding studies; MUC-1 distribution [8,189,193,246,247]). The described local effects of blastocysts on the adjacent uterine epithelium can be studied comfortably in polytocous species (numerous blastocysts) and, in particular, in species with the central mode of implantation like the rabbit. Here, the endometrium of the forming implantation chamber can be compared easily with interblastocyst regions within the same uterus, a fact that has much facilitated the discovery of this phenomenon [244,245].

Locally acting signals appear to be human chorionic gonadotrophin (hCG) and interleukin-1beta (IL1-beta) [248] and may directly affect ULE polarity and other EMT-like processes [10,249]. Research has also been performed on HB-EGF signalling in the mouse [250,251,252,253]. One conclusion was that HB-EGF regulates the implantation process by influencing uterine epithelial cell polarity comprising VANGL2, a key planar cell polarity component [254]. Signalling by the blastocyst may also have a mechanical component that can be mimicked in in vitro models [255]. Much is known about signalling in the large farm animals with the central mode of implantation (ruminants, pig) [1,49,88]. It is an interesting question how much of this information can be translated to species with different implantation types. As far as local actions of these signals are concerned, the large farm animals appear to be a more difficult model than, e.g., the rabbit, another species with the central type of implantation. Due to the more moderate size of the rabbit blastocyst at implantation stages, it is a particularly easy task to compare endometrial parts at the implantation sites with interblastocyst regions, in order to detect signs for local signalling. This does not seem to have been exploited in the same way in the large farm animals, possibly due to the size problems mentioned.

The local stimulation of apical plasma membrane changes in the uterine epithelium may be related to the extrusion of *extracellular vesicles* (*EVs*). EVs have received much attention recently as mediators of message exchange between blastocyst and endometrium. They carry RNAs, proteins, and lipids with signalling functions [254,256,257,258,259,260,261,262,263,264,265], which are protected from extracellular degradation by their inclusion within the EVs. Their target cells have specific docking molecules, and after uptake, the EVs release their contents into the recipient cells. Apical protrusions have been observed at the receptive ULE by electron microscopists for a long time, but their physiological functions remain unclear (“pinopods”, i.e., being considered a kind of fluid transport structures, or indicators of some type of apocrine extrusion [259,266,267,268,269]). It is not clear how they may be related to the shedding of apical plasma membrane markers which includes many enzymes ([187,188], reviewed in [23]). Release of dipeptidyl peptidase IV and aminopeptidase N by human ULE was recently confirmed [270]. Enzymological methods could be used to detect and monitor extrusion of membrane-bound components from the uterine epithelium into the uterine secretion [244,245]. The heterogeneity of the extracellular bodies that are found in the uterine secretion and in the spaces between blastocyst and endometrium still leaves many questions open about their origin and about their specific uptake by cells—whether their extrusion is connected with the transformation of uterine epithelial membranes or cell polarity changes, or with adhesiveness and invasiveness of trophoblast of the implanting blastocyst [271] is still unknown.

An interesting but not widely known special case of local changes is the *epithelial plaque reaction* found in several nonhuman primates. In those species, the uterine epithelium develops during the receptive state a capacity to change its monolayered morphology (simple epithelium) and to become multilayered, i.e., to pile up. This is observed in pregnancy in the immediate vicinity of the implanting blastocyst in Macaques (*Macaca fascicularis* and rhesus monkey), the marmoset, and the baboon [272,273,274,275,276], and it is conceivable that this local reaction is indicative of signalling by the implanting blastocyst. In the rhesus monkey, this response can also be elicited experimentally by traumatization, without the presence of a blastocyst, if a receptive state is induced using an appropriate progesterone treatment [277]. The physiological significance of the epithelial plaque reaction is not clear, but it can serve as an interesting indicator for changes in the expressed programme, i.e., that the new population of cells (the plaque cells) has a down-regulated apico-basal polarity. Piling up of epithelial cells would not be possible with a typical polar organization, i.e., when cells possess a non-adhesive apical plasma membrane (as present in the pre-receptive ULE). The situation is somewhat reminiscent of pre-cancerous states known from other epithelia, where a change from a monolayered organization (with an intact apical plasma membrane) to multilayering (lacking apical plasma membranes) can be observed (see textbooks of pathology, [278]). In these non-human primate models the receptive ULE indicates a capacity for epithelial axis reorganization (upon blastocyst-derived or surrogate traumatic irritation). This further supports the concept that regulations of epithelial polarity are involved in endometrial receptivity, and that a destabilization of ULE polarity may be a key to understanding implantation mechanisms better. Unfortunately, we know little about genes and signalling systems involved in epithelial stratification phenomena in the uterus. A recent study comparing genetic data in the human with knockout experiments in the mouse may be of interest here (knockout of the histone lysine methyltransferase KMT2D which is involved in keratinocyte differentiation), focusing on the implantation failure observed in this mouse model [279]. Other findings in the mouse model are revealing roles for, e.g., the PRICKLE1 gene in the regulation of ULE functional morphology and implantation [280]. Unfortunately, genetic and functional studies are impossible or difficult to perform in primates with their impressive plaque reaction. The plaque was traditionally regarded as some kind of defence reaction against trophoblast invasion and was addressed as an “epithelial variant of a decidual reaction”, a choice of term that, however, should better be avoided because it is confusing [272].

## 6. In Vitro Model Systems: Insights into Functional Aspects

**Figure 6:**
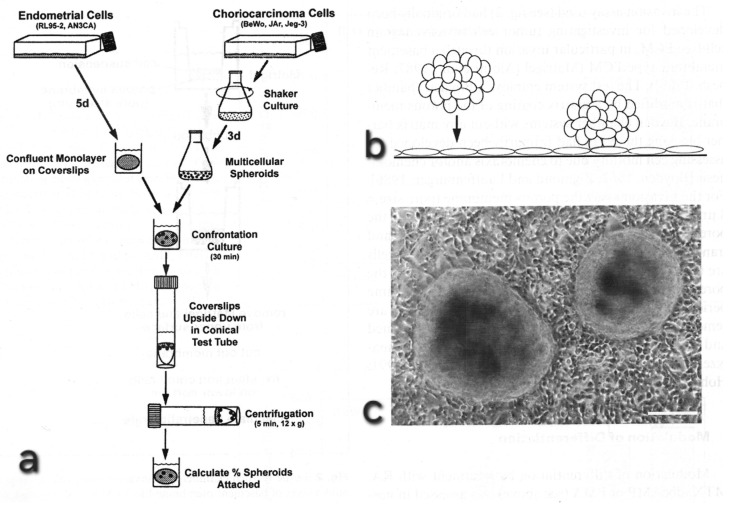
A widely used in vitro assay for model studies on trophoblast type cell attachment to endometrial cells. (**a**) Experimental design (after [281]). (**b**) Sketch of confrontation arrangement (cross section). Multicellular spheroids of trophoblast type cells (e.g., human choriocarcinoma cells like JAr or Jeg-3, above) and endometrial cell monolayer (like RL95-2 or HEC-1A, below). (**c**) JAr cell spheroids adhering to RL95-2 cell monolayer after 30 min of incubation and following centrifugation. Bar: 100 µm (from [65] with permission).

Studying implantation in vivo meets with considerable difficulties even in animal models and is impossible in the human. In vitro systems have been developed to probe for the functional importance of cell polarity and EMT-like phenomena in implantation. In such studies, spare human embryos from IVF-ET clinics have sometimes been used [246] but this not only meets with numerical restrictions but also raises ethical concerns. My group has, for this reason, decided early to refrain entirely from using human blastocysts and to seek developing alternative models. An important aspect was the use of three-dimensionality in the models. For human trophoblast, this aspect was first taken care of by the introduction of the multicellular spheroid model, using initially choriocarcinoma cell lines (BeWo, JAr, Jeg-3) and later, in a similar way, early placenta-derived human trophoblast [68,281,282]. This was inspired by the Mareel assay developed for tumour invasion research [283]. In order to study adhesion to and invasion into endometrium, confrontation models were developed using complex three-dimensional endometrial explants consisting of ULE, glands, and endometrial stroma, first in the rabbit [284,285] and later with human endometria. These endometrial models were confronted with the trophoblast-type cell spheroids that served as blastocyst models [68]. Remarkably, there has recently been a renewed interest in such complex organ-culture type models, e.g., including uteri and blastocysts in the mouse [286]. For studies focusing on cell interaction details of attachment and invasion processes, human trophoblast or choriocarcinoma spheroids were preferably confronted not with the three-dimensional endometrial models but with uterine epithelial monolayers of differing polar organization (Figure 6; cell lines like RL95-2, HEC-1A, and AN3-CA [67,281], or Ishikawa cells which are responsive to steroid hormones [287]). These model systems have in the meantime found wide application in many laboratories, often using the originally proposed cell lines but also various others as well as primary explants [15,115,288,289,290,291,292,293,294,295,296,297,298,299]. Most of the recent variants of such systems (now often called “organoids” or “assembloids”) put emphasis on a three-dimensional organization of the models, and some of these include in vitro reaggregation after starting with dissociated epithelial and stromal cells, or in vitro differentiation from stem cells [300,301,302,303,304]. However, it is important to remember that no in vitro model contains all the cell types present at implantation sites, including cells of immune origin and a functional vascular system.

Already the early variants of these in vitro assays have allowed to show that a polar vs. apolar organization of the ULE model cells is indeed functionally very relevant for trophoblast adhesion, and that their epithelial (vs. dedifferentiated or mesenchymal-like) nature is also important [67,305]), as is the differentiation status of the trophoblast model cells [65,67]. The importance of an epithelial nature has been confirmed by studies on modulation of E-cadherin expression in the originally non-adhesive AN3-CA cells [305,306]. Furthermore, functional tests have been performed, with these assays providing insights into molecular regulations [17,99,305]. With an atomic force microscopy/force spectroscopy approach it was possible to obtain data on the time course of adhesive force development during attachment of trophoblast to uterine epithelial cells [50]. These studies showed that cellular contact formation between the trophoblast and uterine epithelium is a remarkably slow process, indicating that signalling and reorganization processes seem to be involved here and may be the reasons for the slow response observed. The models also allowed to obtain some insights into details of, e.g., calcium signalling [255,307,308]. This appears interesting because ion channel coding gene defects in the endometrium seem to be connected with recurrent implantation failures [309]. These models also provided first insights into the reorganization of the cytoskeleton and the role of regulation by rho proteins [287,310,311]). The dominant conclusion from these model studies was that not only changes in the composition of the apical plasma membrane are important but that a destabilization of the entire apico-basal organization of the ULE is a prerequisite for trophoblast attachment and invasion, and that reorganization of the cytoskeletal system is involved. The process of reorganization of the cell architecture and functionality seems to continue after initial apical attachment, finally allowing trophoblast penetration into and through the ULE, which can be investigated well in these in vitro systems [13,37,240,287,310].

## 7. Conclusions

In conclusion, it now appears that the ULE is not only a passive partner (as may be suggested by terms like “receptivity” and “permissiveness”) but participates actively in implantation of the embryo [17,287,310,311]. At receptivity the apical pole of the ULE is predisposed for an interaction with trophoblast, in contrast to other cell types. This predisposition includes the ability to transmit various (including mechanical) signals to the cell interior, including in-the-transmission machinery, e.g., membrane-bound integrins and an appropriately rearranged actin cytoskeleton. The formation of adhesion of trophoblast to a ULE model in vitro was recognized as a relatively slow process, probably including complex signal transduction cascades and sequential steps of bond formation [50]. By extrapolation, the process of blastocyst interaction with the ULE in utero, with initial arrest followed by firm implantation, may involve a complex transition from weak to strong binding and in many species by penetration, perhaps in a way that is somewhat similar to the rolling, arrest, and penetration cascades in leukocyte–endothelial cell interactions (reviewed in various contributions in [312].

Based on these observations, the receptive state of the endometrium was re-defined as a specific condition of this tissue in which the ULE is the critical effector taking on, for a limited hormonally controlled time period, peculiar cellular properties, not met by epithelia of other organs [96] (but regulated itself by epithelial–stromal interactions). Initiation of embryo implantation requires that trophoblast and ULE down-regulate part of their epithelial-type differentiation programme in a controlled way. The ULE destabilizes its apico-basal polarity. In this way this epithelial lining acquires the peculiar property to become responsive to locally acting signals from the trophoblast which cause a continuation of structural and functional reorganization of the ULE, completion of the state of permissiveness for apical attachment, and (in most species) for subsequent invasion by trophoblast cells. Initially, major changes involve the apical plasma membrane domain which reduces its repellent properties and acquires an adhesion competence which is specific for certain activated trophoblast-type cells [99] (not, e.g., invasive non-trophoblast tumour cells). This change in functional organization occurs, however, not only at the apical cell pole—the transformation to receptivity is connected with multiple changes in cell structure and functions, not restricted to the plasma membrane. The way how, in the receptive state, uterine epithelial cells become responsive to chemical (and probably also mechanical) signalling from the trophoblast, in the sense that they can complete their receptivity, still needs to be studied much more in detail. Consequently, the signalling processes that must play a role here are of particular interest. They are now under intense investigation, making use of various developed in vitro systems discussed above. Our knowledge about the involved structural and functional changes that are elicited as a result is still limited. Functional assays are very important in order to gain insights into the nature of the cell changes, and in vitro models are of great importance here.

The EMT concept has been helpful for channelling attention to the role of more general changes in the physiological states, in contrast to selective protein expression or membrane composition changes, in preparation of trophoblast and ULE for implantation initiation. However, ULE cells (and likewise trophoblast cells) do not convert completely to a mesenchymal phenotype [6,96], as a strict application of the EMT concept would suggest. Indeed the “hybrid” nature, as shown by the expressed programme of the uterine epithelium at receptivity, may be a key feature that endows the endometrium with a delicate regulatory role in order to keep trophoblast attachment and invasion a very local phenomenon [99]. It was necessary to take more detailed views into changes in cell organization and signalling processes to arrive at the conclusion that the uterine epithelium participates actively in adhesion of trophoblast and in permitting its penetration, rather than remaining passive [17,287,310,311]. This necessity for an active participation of the host tissue is certainly one factor that, together with local signalling by trophoblast, ensures restriction of the invasive interaction locally and in a strictly controlled way. This seems to be achieved via maintaining, during the epithelial penetration phase, major epithelial properties in the trophoblast as well as the ULE as discussed above. It is by this way that an “embryo penetration route” [13] becomes defined and locally restricted at a cellular level.

**Figure 7:**
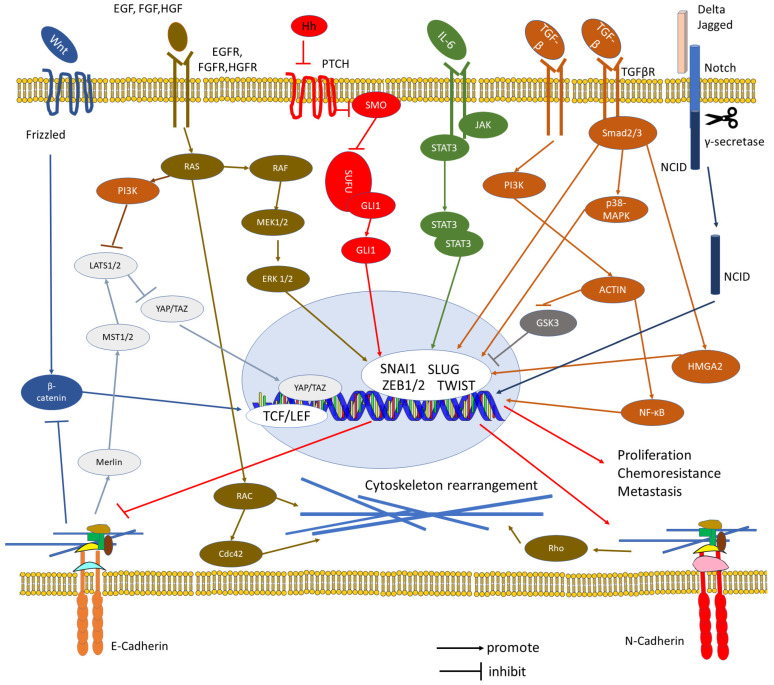
A recent summary of signalling pathways involved in epithelial–mesenchymal transitions (EMT) emphasizing crosstalks between pathways (reprinted from [30]). Crosstalk between multiple signalling pathways increases the expression of EMT transcription factors including SNAI1, SLUG, TWIST, and ZEB, leading to the loss of epithelial characteristics and gain of mesenchymal characteristics. Wnt/β-catenin pathway induces EMT by interacting with TCF/LEF. As discussed in the present text, available data suggest that endometrial receptivity and trophoblast attachment competence/invasiveness involve only parts of an EMT-like programme. Of special interest for ongoing research is, therefore, which of the various EMT signalling pathways, or any others instead, are playing a role in implantation and how.

Still, the exact nature of the peculiar states of permissiveness (receptivity) of the uterine epithelium for trophoblast adhesion and of attachment competence and invasiveness of the trophoblast leaves many detailed questions open. It is clear that transition to these states requires relatively global changes in many aspects of epithelial cell organization, but not a complete switch to a mesenchymal cell state as an application of the original EMT concept would suggest. This view is now increasingly being accepted, e.g., in the interpretation of data on the roles of the HOXA10 gene and the Twist2 transcription factor (some authors now preferring the term “partial EMT” [16]). The molecular details of such changes, as well as of the signalling processes involved, are under scrutiny in a number of laboratories, as is their regulation by ovarian steroids and embryonic stimuli [12,15,16,26,30,93] (Figure 7; see also contributions to this SI of *Biomolecules* [24]). Conceptionally, novel approaches revealing that EMT can be subdivided into several intermediate cell states, characterized by special subgroups of gene regulatory networks and thus providing much flexibility [243], may open ways how to modernize and to specify in molecular terms the original proposal that endometrial receptivity makes use of specific parts, but only parts of EMT-like programmes.

For a real understanding of the mechanisms behind implantation, and for any attempts at applying this knowledge for clinical purpose, we will need to know which parts of the whole spectrum of elementary processes (as addressed in the EMT concept) are decisive for endometrial receptivity and its completion in the implantation chamber due to blastocyst signalling as well as for trophoblast invasiveness. It can still be beneficial to take lessons from the related fields of tumour cell invasion and embryonic fusion processes that have been addressed above. Since the aspect of *partial* EMT has become a topic in tumour research [27,28], it may be of interest to compare what is known these days about the whole spectrum of signalling systems involved in EMT (Figure 7 [30]) with what is indeed found in tumour cell invasion, in development, and in processes like wound healing [28,29,128,242,313,314,315,316,317], and to ask what may be specific in endometrial receptivity and interactions between ULE and implanting trophoblast. Such a comparison may be helpful for the further planning of implantation research. Specifically, recent attempts at tumour drug development could be of interest for basic research in implantation since they could provide research tools (for recent developments in targeting EMT in tumour research and therapy approaches see, e.g., [318]). The impressive accumulation of new molecular and cell physiological data that have been obtained on endometrium and trophoblast in the years since the implantation paradox was first described may now open a chance to arrive at a better understanding of the cell biological basis of these transitions in cell states and of the signalling processes involved. Some of the factors that have been put into focus by the concepts of epithelial cell polarity, adhesion, and EMT are now indeed receiving increasing attention in attempts at improving success rates in ART practice, relating them to endometrial receptivity problems (e.g., the role of PDX, HOXA10/11 [319], or influencing EMT-related processes via manipulating E-cadherin expression using EVs and miRNA, based on research conducted with the in vitro systems discussed above [306]). The present Special Issue of *Biomolecules* [24] intends to provide a source of information about such a *tour d’horizon* in the field of implantation research and a stimulus for rethinking the concepts.

## Figures and Tables

**Table 1:** Examples of embryonic “fusion” processes which start with apical cell–cell contacts of opposing epithelia (comparable to implantation) (data from [5,96], updated with more recent references).

Type of Epithelium and Fusion Process	References
***Various epithelia***	
Neural tube closure (combined with formation and emigration of neural crest cells)	[127,128,129,130,131,132,133,134,135,136,137,138,139,140,141,142,143]
Otic vesicle closure	[144,145,146]
Semicircular canal formation	[147,148,149] (Figure 2)
Lens vesicle closure	[150,151,152,153,154]
Secondary palate (fusion of palatal shelves)	[130,152,155,156,157,158,159,160,161,162,163,164,165,166] (Figure 3)
Nasolacrimal duct formation	[167,168]
Fusion of nasal processes	[130,165,169]
***Mesothelium***	
Closure of the pleuro-peritoneal canal at formation of the diaphragm	[130,170,171]
***Endothelium***	
Fusion of endocardial cushions during septation of the heart	[130,172,173,174,175,176,177,178,179]

**Table 3:** Cellular and molecular characteristics originally proposed as landmarks for the epithelial vs. mesenchymal phenotype, and for indicating EMT processes (based on [34,35,36,165,220,221,222,223,224]).

Epithelial Phenotype	Mesenchymal Phenotype
**STRUCTURAL ORGANIZATION** ***General*** Apico-basal polarity ***Plasma membranes*** ***Apical plasma membrane (apm):*** Specialized glycocalyx Typical apm proteins including apical marker enzymes ***Basolateral plasma membrane:*** Lateral: junctional complex and associated proteins (adherens junctions: E-cadherin; occludens junctions: occludin; gap junctions: connexins) Basal: adhesion to basement membrane (BM) adhesion complex (integrin alpha 6 beta 4; hemidesmosomes)	**STRUCTURAL ORGANIZATION** ***General*** Front-rear end polarity, filopodia ***Plasma membranes*** Matrix (ECM) interaction proteins (integrin alpha 5 beta 1, integrin alpha V beta 3)
***Cytoskeleton*** Cytokeratins	***Cytoskeleton*** Vimentin
**ECM PROTEIN PRODUCTION** Collagen type IV, laminin (basement membrane, BM)	**ECM PROTEIN PRODUCTION** Collagen type I, fibronectin
**CELL BEHAVIOUR** Homotypic cell–cell adhesion, cell sheet formation Sedentary Movement as cell sheets Specialized adhesive interaction with basement membrane at basal cell pole	**CELL BEHAVIOUR** Cell–matrix (ECM) interaction Individual movement and invasion possible (production of ECM-de- grading enzymes)

## Data Availability

No new data were created or analyzed in this study. Data sharing is not applicable to this article.
